# Lycopene: A Natural Arsenal in the War against Oxidative Stress and Cardiovascular Diseases

**DOI:** 10.3390/antiox11020232

**Published:** 2022-01-26

**Authors:** May Nasser Bin-Jumah, Muhammad Shahid Nadeem, Sadaf Jamal Gilani, Bismillah Mubeen, Inam Ullah, Sami I. Alzarea, Mohammed M. Ghoneim, Sultan Alshehri, Fahad A. Al-Abbasi, Imran Kazmi

**Affiliations:** 1 Biology Department, College of Science, Princess Nourah Bint Abdulrahman University, Riyadh 11671, Saudi Arabia; mnbinjumah@pnu.edu.sa; 2 Environment and Biomaterial Unit, Health Sciences Research Center, Princess Nourah Bint Abdulrahman University, Riyadh 11671, Saudi Arabia; 3 Department of Biochemistry, Faculty of Science, King Abdulaziz University, Jeddah 21589, Saudi Arabia; fabbasi@kau.edu.sa; 4 Department of Basic Health Sciences, Preparatory Year, Princess Nourah Bint Abdulrahman University, Riyadh 11671, Saudi Arabia; SJGlani@pnu.edu.sa; 5 Institute of Molecular Biology and Biotechnology, The University of Lahore, Lahore 54000, Pakistan; bismillah.mubeen@gmail.com (B.M.); inamgenetics@gmail.com (I.U.); 6 Department of Pharmacology, College of Pharmacy, Jouf University, Sakaka 72341, Saudi Arabia; samisz@ju.edu.sa; 7 Department of Pharmacy Practice, College of Pharmacy, AlMaarefa University, Ad Diriyah 13713, Saudi Arabia; mghoneim@mcst.edu.sa; 8 Department of Pharmaceutics, College of Pharmacy, King Saud University, Riyadh 11451, Saudi Arabia; salshehri1@ksu.edu.sa

**Keywords:** lycopene, nutraceutical, reactive oxygen species, coronary artery disease, hypertension

## Abstract

Lycopene is a bioactive red pigment found in plants, especially in red fruits and vegetables, including tomato, pink guava, papaya, pink grapefruit, and watermelon. Several research reports have advocated its positive impact on human health and physiology. For humans, lycopene is an essential substance obtained from dietary sources to fulfil the body requirements. The production of reactive oxygen species (ROS) causing oxidative stress and downstream complications include one of the major health concerns worldwide. In recent years, oxidative stress and its counter strategies have attracted biomedical research in order to manage the emerging health issues. Lycopene has been reported to directly interact with ROS, which can help to prevent chronic diseases, including diabetes and neurodegenerative and cardiovascular diseases. In this context, the present review article was written to provide an accumulative account of protective and ameliorative effects of lycopene on coronary artery disease (CAD) and hypertension, which are the leading causes of death worldwide. Lycopene is a potent antioxidant that fights ROS and, subsequently, complications. It reduces blood pressure via inhibiting the angiotensin-converting enzyme and regulating nitrous oxide bioavailability. It plays an important role in lowering of LDL (low-density lipoproteins) and improving HDL (high-density lipoproteins) levels to minimize atherosclerosis, which protects the onset of coronary artery disease and hypertension. Various studies have advocated that lycopene exhibited a combating competence in the treatment of these diseases. Owing to all the antioxidant, anti-diabetic, and anti-hypertensive properties, lycopene provides a potential nutraceutical with a protective and curing ability against coronary artery disease and hypertension.

## 1. Introduction

Bioactive components can be found in plant-based natural products derived through food processing [1]. Many of these plant metabolites aid in the reduction of oxidative stress, making them potentially useful in the treatment of a wide range of severe illnesses. Despite the availability of numerous medications to treat oxidative stress-related chronic diseases, the high profile of drug side effects necessitates the use of alternative and complementary treatment options for diabetes and cardiovascular diseases (CVDs), such as coronary artery disease (CAD) and blood pressure control [2,3]. To avoid or treat chronic disorders, lifestyle adjustments and dietary interventions, such as increasing fruit and vegetable consumption, are frequently advocated [4,5,6]. Lycopene is a red-colored compound found in colored fruits and vegetables, including tomato, papaya, pink guava, and watermelon, and is responsible for their reddish hue. Tomatoes and tomato-based products are the most common sources of lycopene [7,8]. Tomato sauce and ketchup are better sources of sources of lycopene as compared to natural raw tomatoes [9]. Lycopene is a natural substance that may be used in high doses as a dietary supplement without causing harm to human health or physiology [10,11,12]. In accordance with these findings, lycopene has gotten a lot of interest as a possible nutraceutical for disease prevention and therapy, notably for improving vascular function and lowering blood pressure [13,14,15]. Biological and biomedical researchers are becoming increasingly interested in the expanding body of evidence indicating lycopene’s disease-preventive properties. Lycopene-rich diets have been inversely associated with heart diseases and malignancies by several in vitro, ex vivo, and in vivo studies [16,17,18]. The buildup of ROS, which is accompanied by abnormalities such as inflammation and irregular lipid metabolism, is a critical risk factor for the increasing occurrence of metabolic disorders [19,20]. ROS, also known as free radicals, are highly reactive, unstable oxygen-containing molecules that can cause cell death by damaging deoxyribonucleic acid (DNA), ribonucleic acid (RNA), and proteins [21,22]. The fundamental biological function of lycopene is the protestation of DNA from oxidative stress by quenching ROS and inhibiting mutations that might cause chronic diseases [23]. The elongated carbon chain with conjugated double bonds have made lycopene as the most potent single oxygen and free radical scavenger among 600 naturally occurring carotenoids [24,25,26]. It is more efficient in shielding cells and tissues from ROS-induced damage [9,27].

**Figure 1:**
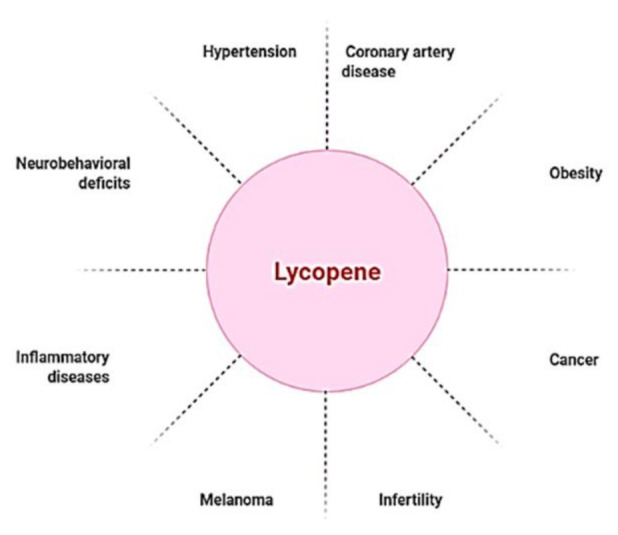
Lycopene as a nutraceutical compound has applications against multiple diseased conditions.

Though lycopene is beneficial for a range of ailments (Figure 1), it is especially useful in the treatment of cardiovascular diseases (CVDs), the leading cause of mortality globally. CVDs are exacerbated by high blood pressure, high cholesterol, and smoking [28,29]. Blood flow to the heart and central nervous system is often restricted, resulting in arterial remodeling and atherosclerosis, which are the principal causes of coronary artery disease [30]. Hypertension, one of the most common causes of cardiovascular morbidity and death, is caused by a restriction of blood flow caused by modified arteries. As a result, hypertension and coronary artery disease have a strong and frequent relationship [31]. A number of pathophysiologic pathways are shared by both disorders. Endothelial dysfunction, which aggravates atherosclerosis and makes atherosclerotic plaques more unstable, is caused by hypertension [32,33,34]. Several studies on lycopene supplementation have showed promising results in lowering blood pressure and coronary artery disease [35,36]. There were no harmful effects at high lycopene consumption levels, according to safety evaluation studies [37,38]. Because lycopene is a lipid-soluble antioxidant, cholesterol-lowering drugs, such as probucol and cholestyramine, diminish lycopene blood concentrations, owing to gastrointestinal absorption issues [39].

Since natural medications are being given increasing attention due to the widespread usage of natural chemicals rather than synthetic drugs to treat ailments, and lycopene is a promising nutraceutical in treating a variety of diseases by blocking disease pathways. This review has focused on the potential effects of lycopene on coronary artery disease and hypertension.

## 2. Discovery, Chemical Structure, Properties, Biosynthesis and Physiological Role of Lycopene

**Figure 2:**
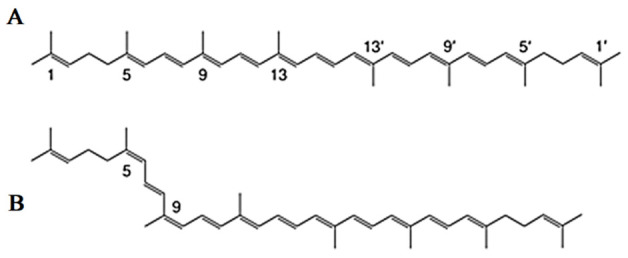
Common isomers of lycopene. (**A**) all-E-lycopene isomer, (**B**) Z-lycopene isomer.

Lycopene is a carotenoid with a molecular formula (C40H56) that gives the *Solanum lycopersicum* L. fruits their red color [40]. Chemically, the lycopene molecule has 11 conjugated double bonds, and its structures can have over 70 Z-isomers [41,42]. According to estimations, all-E-lycopene contains 80 percent to 97 percent lycopene in tomato fruit. However, Z-isoforms account for more than half of the lycopene present in human blood and tissues [43,44] (Figure 2).

Millardet identified lycopene in 1876 and named it as ‘soanorubin’; later on, it was named lycopene and it was purified [45]. Tomato is a major source of natural lycopene; however, it is found in many plants at variable concentrations [46,47]. The extended conjugated double bond system of these compounds is a significant property of the carotenoids and is responsible for their attractive colors [48,49]. Lycopene produces the light-absorbing chromophore, and the extended conjugated double bond system of these compounds is a significant property of the carotenoids that is responsible for their attractive colors. In order for a molecule to have visible color, it must have at least seven conjugated double bonds. The maximum absorption wavelength increases as the number of conjugated double bonds increases [50,51]. Lycopene is not a precursor molecule for vitamin A because it lacks the terminal b-ionic ring found in vitamin A’s core structure. Lycopene is the most effective singlet oxygen quencher among the carotenoids, with the number of conjugated double bonds and, to a lesser degree, the presence of cyclic or acyclic end groups dictating its quenching ability [52]. Furthermore, its biological properties, such as oxidative sensitivity, are impacted by its chain structure, which includes a large conjugated polyene system [53,54]. Lycopene is found in nature as an all trans form with seven double bonds that can be isomerized to mono-cis or poly-cis when exposed to high temperatures, light, oxygen, acids, catalysts, and metal ions. Lycopene is a lipophilic molecule with hydrophobic properties due to its acyclic structure and 11 linear conjugated double bonds, making it more soluble in organic solvents, such as chloroform, benzene, hexane, methylene chloride, acetone, and petroleum ether [55]. Lycopene is a vivid red pigment that is water insoluble [56]. Lycopene is present in the chloroplasts of fresh fruit, a plant cell organelle that is rarely eaten [57]. [57]. Thermal food processing, particularly in the presence of cooking oils, causes lycopene to micellize and enhance its intestinal absorption rate by a factor of ten [58].

**Figure 3:**
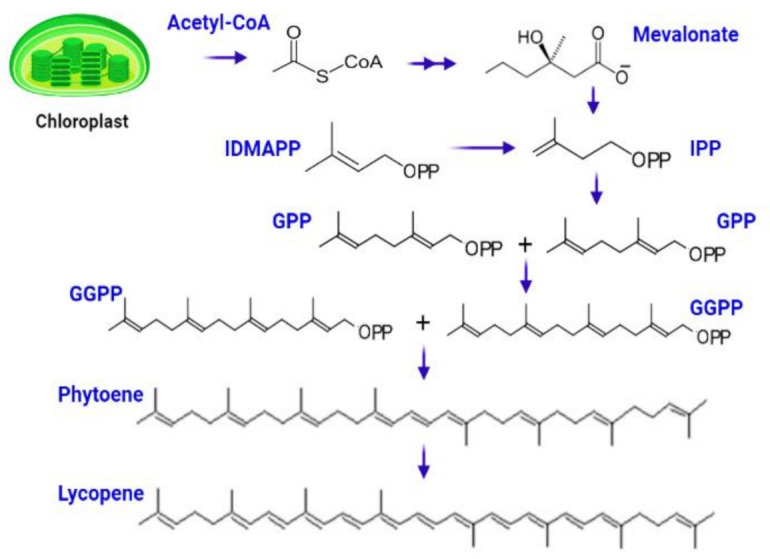
Biosynthesis of lycopene starts from central metabolite Acetyle-co-A, which is subsequently converted to IPP, GPP, GGPP, phytoene, and lycopene.

The complicated process of lycopene manufacturing begins when chlorophyll degrades to produce white-colored leucoplast, which produces particular red-colored pigmented organelles called chromoplast. The biosynthetic process begins with the conversion of acetyl-Co-A to isopentenyl diphosphate (IPP) via the mevalonate route [59]. IPP (5C) interacts with DMAPP (5C) to form geranyl diphosphate (GPP), a ten-carbon molecule [60]. The next step entails adding two IPP molecules one at a time, resulting in the synthesis of geranylgeranyl diphosphate (GGPP), a 20-carbon complex. Two molecules of GGPP are joined head-to-head in a condensation process to generate phytoene, a 40-carbon chemical that is then converted to lycopene via a mechanism mediated by phytoene desaturase [61,62] (Figure 3).

The brilliant crimson hue of lycopene crystals in the shape of small globules hang throughout the fruit [63]. Lycopene is located in the thylakoid membranes as a protein lycopene complex at the cellular level, owing to its lipophilic nature. Despite the fact that lycopene is not an essential ingredient, it has been discovered to provide a variety of health advantages. Because it is a substantial carotenoid in human blood, it can protect lipids, proteins, and DNA from oxidative stress [21]. Lycopene’s antioxidant activity may be enhanced by the absence of the b ionone ring structure. Due to stereochemical variations, lycopene differs from other regularly eaten carotenoids in that it can only be found in particular subcellular locations. The human body directly absorbs a substantial amount of intact lycopene, which circulates through the body’s plasma, liver, and peripheral organs. It accumulates in the human tissues but is not evenly distributed [64]. Adipose tissues, adrenal glands, testes, and liver, for example, have larger concentrations, while the kidneys, prostate, lungs, and ovaries have lower concentrations [56]. Plasma lycopene has a half-life of 12–33 days in the human body [65]. Lycopene must be absorbed and incorporated into the plasma and tissues in order to be used as a dietary supplement. Tomato lycopene is not easily absorbed since it is integrated into the nutritional matrix. Clinical research demonstrates that heat-processed tomato products absorb lycopene more quickly than raw sources, and that adding oil increases absorption [66].

**Figure 4:**
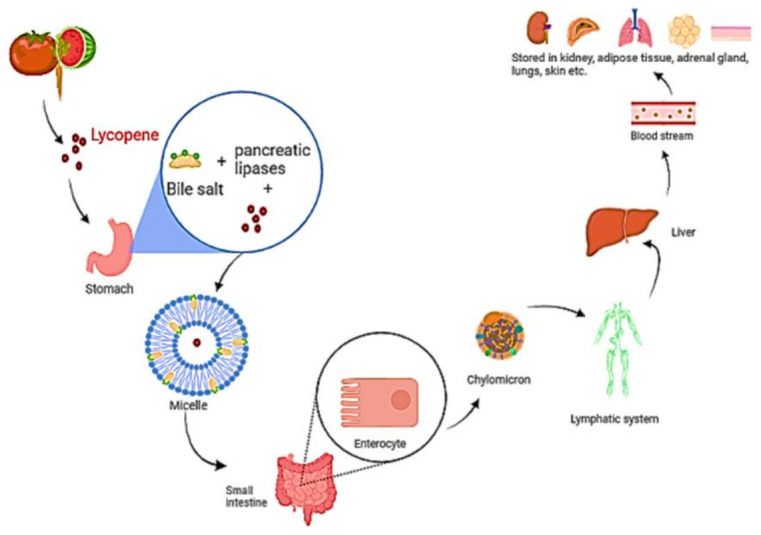
A diagram depicting the digestion and absorption of lycopene. Food releases lycopene, which is then integrated into micelles containing bile salt, cholesterol, and fatty acids. The micelle approaches enterocytes, and lycopene diffuses over the apical membrane in a passive manner. Lycopene is packed with other dietary lipids inside the enterocyte to form chylomicrons, which are carried over the basolateral membrane, into the lymphatic system, and subsequently discharged into the blood.

Isomerization of lycopene at low pH in the stomach has been described [67]. Before the lycopene is integrated into mixed micelles, it must be freed from the food matrix. Micelles include bile salts, cholesterol, and fatty acids from the meal and their amphiphilic shape aids in keeping the lipophilic nutrients soluble in the watery digesta [68]. The micelles approach the apical side of intestinal enterocytes’ unstirred water layer, where lycopene diffuses passively over the apical membrane [69]. Lycopene is considered to be absorbed in the same way that dietary lipids are, via passive diffusion [70,71]. According to research, the scavenger receptor class B type I (SR-BI) cholesterol membrane transporter aids in lycopene absorption. It has also been observed that various additional transporters are linked to lycopene absorption. [72,73]. Once within the enterocyte, lycopene is bound with dietary lipids to form chylomicrons [69], which are then transported via the basolateral membrane, into the lymphatic system, and finally discharged into the circulation (Figure 4). Lycopene transport and distribution are aided by plasma lipoproteins. It continues to be found in the lipophilic area of lipoproteins, which is the hydrophobic molecule’s core [18,74]. Lycopene is mostly transported by low-density lipoproteins [75,76]. Furthermore, cis isomers of lycopene have been found to have a stronger capacity to integrate into lipoprotein and other proteins than all trans isomers, owing to their shorter chain length [77].

## 3. Lycopene as an Antioxidant

A stressor is any agent that induces stress [78,79]. Stress is an organism’s general response to negative stimuli. Stress affects the physiological balance by causing a biological reaction to stimuli [80]. Oxidative stress, which is induced by highly reactive free radicals, is one of the primary causes of chronic illness [81,82]. Antioxidants have been identified as a varied set of chemicals that inhibit oxidation in various ways [83,84,85,86]. Only a few lipophilic natural oxidants exist, and lycopene is one of them. As a powerful singlet oxygen quencher, it can stop lipid oxidation in its early stages. Lycopene’s ability to protect against oxidative stress has been established [87,88]. Lycopene has been found to be more potent in this activity as compared to other carotenoids, such as tocopherol, ß-cryptoxanthin, carotene, lutein, and zeaxanthin [9]. B-carotene and a-tocopherol, two more lipophilic antioxidants, had double and 100-fold lower rates, respectively [89]. Lycopene’s major biological purpose is to protect DNA from oxidative stress in order to prevent mutations that might lead to chronic diseases [90,91,92]. ycopene is the most potent free radical and single oxygen scavenger among 600 naturally occurring carotenoids because of its long chain with conjugated double bonds [93,94]. It modulates phase I and II detoxifying enzymes, which affect cell proliferation, immunological response, and gene transcription [24]. It activates the antioxidant response element (ARE), which causes the cellular enzymes glutathione S-transferase (GST), superoxide dismutase (SOD), and quinone reductase to be synthesized [95,96,97,98]. HO-1, GST, NQO1, and SOD are antioxidant and detoxifying enzymes that are sometimes referred to as phase II cytoprotective enzymes [46,99]. ARE is located in the promoter regions of inducible genes that code for phase II enzymes, and it promotes overexpression of these genes when it binds to Nrf2. Lycopene inhibits Nrf2/Keapl binding in heat stressed birds, allowing Nrf2 to be transported to the nucleus and upregulate phase II enzyme synthesis [100].

The electrophile response element transcription system (EpRE) or antioxidant response element transcription system (ARE) are related to the cis-regulatory portions in the promoter region of detoxifying enzymes [26,101]. By activating the ARE transcription pathway [26], lycopene can affect xenobiotic metabolism by disrupting the cytosolic linkages between the major ARE-activating Nrf2 and its inhibitor (Keap1) [26,102]. Once liberated of Keap1, Nrf2 translocates to the nucleus, where it induces phase II enzyme expression [26,103]. According to some research, overexpression of phase II detoxification enzymes, in addition to blocking phase I metabolism and metabolic activation of aflatoxin B1, enhances lycopene’s anti-aflatoxin actions [104,105]. Lycopene operates in three methods to produce reactive oxygen species (ROS): first, radical addition (adduct formation), then electron transfer to the radical, and finally, allylic hydrogen abstraction [106]. Two processes that contribute to lycopene’s antioxidative impact are the formation of adducts and allylic hydrogen abstraction [107,108]. The type of the reacting free radical, the structural characteristics of lycopene, and the positioning and direction of lycopene inside the membrane in biological systems are all elements that impact these potential interactions [109,110,111].

The non-polarity of cell membranes or micelles is aided by the polar environment, which assists in the production of adducts and allylic hydrogen abstraction [107]. Lycopene and free radical reactions can occur in a variety of ways at the same time [109]. Lycopene can increase the cellular antioxidant defense system by regenerating non-enzymatic antioxidants, such as vitamins E and C, from their radicals [26]. Vitamin E is suggested to be protected by lycopene [87,112,113]. Lycopene serves as an antioxidant in systems that create singlet oxygen, but as a pro-oxidant in systems that generate peroxide. Lycopene serves as an antioxidant due to its redox potential [114,115,116]. Indeed, lycopene behaves as a pro-oxidant in high doses while acting as an antioxidant in low ones [117]. Many factors impact pro-oxidant potency, including tissue oxygen tension, lycopene concentration, and interactions with other antioxidants [115]. As a pro-oxidant, lycopene may have both good and negative impacts in biological systems, as well as influence the course of human illnesses. If lycopene works as a pro-oxidant in previously damaged cells, it may help prevent the creation and progression of cancerous lesions as well as tumor cytotoxicity. Carotenoids’ pro-oxidant effects can be limited by antioxidant connections, enhancing the antioxidant capabilities of these bioactive molecules [115].

## 4. Lycopene in Human Health and Diseases

Humans benefit from lycopene in a number of ways [26]. A lycopene-rich diet may help to prevent or lower the risk of cardiovascular disease and some malignancies [118]. According to study [119], 5 to 7 mg of lycopene per day may be sufficient to gain the benefits. Higher doses of lycopene (35–75 mg/day) may be provided in the occurrence of cancer or cardiovascular disease [120]. When combined with prostaglandins and phospholipids in cell membranes, lycopene can improve skin defense mechanisms [121]. Lycopene has been linked to the prevention and treatment of a wide range of ailments (Table 1).

## 5. Lycopene in Cardiovascular Diseases (CVDs)

CVDs are the leading cause of illness and death around the world. High blood pressure, cholesterol, and smoking are all major risk factors for cardiovascular disease. Damage and remodeling of blood vessels impede blood flow, and atherosclerosis is the most prevalent cause of CVDs, which affect the heart and brain [156]. In comparison to Mediterranean nations, Europe and the United States have the greatest rate of CVDs [157]. The lower rates of CVD have been related to a diet heavy in vegetables, such as tomatoes, tomato derivatives, and olive oil. Small concentrations of lycopene in the blood, on the other hand, have been associated to hypertension, myocardial infarction, stroke, and atherosclerosis. Lycopene concentrations in the blood have been proven to reduce the risk of serious cardiac events. [158]. Epidemiological studies strongly advocate the preventive role of lycopene in CVDs. Low blood lycopene levels have been linked to all-cause mortality and poor cardiovascular disease outcomes. Lycopene supplementation has been shown to increase blood lycopene levels, reduce oxidative stress markers, and improve antioxidant status [76]. Reduction in the pro-inflammatory cytokines, adhesion molecules, inhibition of leukocyte migration and inflammation-related genes, problems in the interaction of monocyte with endothelium, activation of T-lymphocytes, and cyclooxygenase-2 downregulation are all anti-inflammatory mechanisms. Lycopene inhibited TNF-induced NF-kB activation and monocyte-endothelial cell interaction [159]. VCAM-1 and LDL were found inversely linked to serum lycopene [160]. Supplementation with lycopene can improve microvascular function by lowering sVCAM and sICAM concentrations, reducing DNA damage, and increasing superoxide dismutase (SOD) activity [161,162,163]. Advanced glycation end products (AGE) include a diverse group of adducts generated by the glycoxidation or glycation of DNA or protein molecules by reducing sugars [164]. Interaction of AGEs with their corresponding receptors RAGEs is the major cause of several disorders. AGE and RAGE interaction initiates oxidative stress via several pathways, such as activation of NF-kB, upregulation of gene expression for cytokines, and stimulation of NOX enzymes. Oxidative stress causes several diseases, including renal failure [165,166]. The reduction in NO production, impairment of endothelium, increased rate of mRNA degradation, neurodegenerative diseases, damage to blood vessels, and diabetes are some other subsequent effects of oxidative stress [167,168]. In this context, the natural or synthesized molecules with the potential to inhibit or reduce the production or interaction of AGEs and RAGEs have a great significance in current biomedical research. According to research reports, lycopene can reduce the production of AGE and RAGE, which aids in vessel protection [6,137,169]. The use of lycopene can promote the function of endothelial cells, as indicated by preclinical studies. Lycopene has the ability to improve the NO bioavailability, endothelium-regulated vasodilation [170], reduce the damage to proteins, DNA, and lipids, and improve mitochondrial functioning, through its antioxidant activity [171]. Lycopene supplementation boosted mitochondrial gene expression and lowered mitochondrial dysfunction [6,172]. Lycopene and tomato products were found to decrease the total cholesterol and low-density lipoprotein cholesterol (LDL-C) in clinical investigations [173,174,175]. In healthy postmenopausal women, lycopene supplementation can decrease total and LDL cholesterol [176]. In rats given lycopene supplements [177], HDL was increased significantly and LDL, triglycerides, and total cholesterol were decreased. A significant decrease in TG in lycopene-supplemented hamsters [178] and a reduction in oxidized LDL in lycopene-supplemented rats have also been reported [179]. IMT is a well-established biomarker of arterial structural change [180], and it has been associated to the presence of cardiovascular risk factors, notably in the carotid artery [181]. The thickness of the intima-media is inversely linked to serum lycopene levels [182]. The combination of lutein and lycopene (20 mg each) therapy resulted in a decrease in IMT after 12 months, with the combination showing to be more helpful than lutein alone.

### 5.1. Coronary Artery Disease (CAD) and Lycopene

Cardiovascular diseases with more that 17 million deaths every year include one of the major causes of death worldwide. CAD, which has become almost epidemic in many societies, is the most prominent CVD [183]. It is a chronic inflammatory disease caused by the remodeling of coronary arteries due to the narrowing of internal passage and hardening of vessels by plaque formation [184,185]. In addition to the above, the activation of platelets and inflammatory factors also contribute to reduce the blood flow to the heart muscles, reducing the supply of oxygen and nutrients [186], especially during vigorous exertion. Atherosclerosis is a silent and gradual process demonstrated by the accumulation of low-density lipids and inflammatory factors in the arteries [187,188,189,190]. Oxidized low-density lipoprotein cholesterol LDL-C is the main contributor to the development of atherosclerosis and subsequent CAD via the activation and differentiation of monocytes to macrophages [191]. Macrophages interact with LDL-C, and the production of interleukins, cytokines, and tumor necrosis factors is induced. All these molecules contribute to the formation of first lesion of atherosclerosis [192,193]. The smooth muscle cells are migrated to the intima from the medial layer of the artery, leading to the formation of a fibrous cap over the streak of lipid materials and subsequent formation of second lesion of atherosclerosis (plaque) [194]. The nature of the fibrous cap determines the properties of the plaque. Stable plaque is composed of an intact cap of smooth muscle cells in combination with collagen type I and III. Such a type of plaque results in stenosis, reduces the blood flow, and results in ischemia [195]. The second type of plaque, thin vulnerable plaques, is made up of type I collagen in combination with only a few smooth muscle cells. However, it contains a major proportion of proinflammatory molecules, macrophages, and prothrombotic molecules [196,197]. These plaques can erode or rapture, while the coagulatory proteins circulating in the blood can interact with them, resulting in thrombosis and acute coronary syndrome [198,199]. Chronic CAD can cause heart failure [200], altered heart rate [201], myocardial ischemia, and left ventricular dysfunction [202]. In epidemiological research, the risk pattern for coronary artery disease has been thoroughly investigated. Age, male sex, raised LDL-C levels, low HDL-C levels, diabetes mellitus, food, genetics, and cigarette smoking are prominent risk factors for coronary heart disease [203,204,205,206]. Obesity and metabolic disorders are also considered as the risk factors for CAD [36,207].

**Figure 5:**
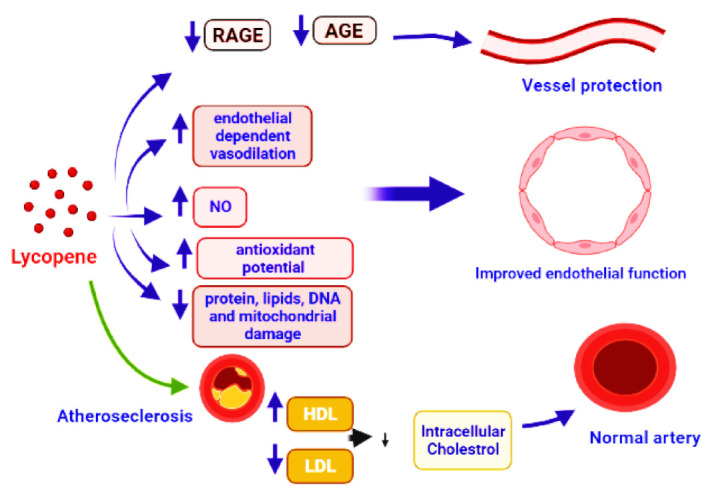
Demonstration of vessel protection by the application of lycopene that caused the inhibition of AGE and RAGE production. It also leads to vasodilation, protection of proteins, DNA, lipids, and mitochondrial damage, by boosting the antioxidant activity. Improved endothelial function is reached by increasing nitric oxide (NO) bioavailability, ensuring positive changes in the lipid profile and healthy arteries, and preventing atherosclerosis.

Lycopene suppresses the formation of vascular smooth muscle cells (VSMCs) and foam cells, both of which have anti-atherosclerotic characteristics [208,209,210]. Contractile VSMC change into proliferative and migratory cells during the atherosclerotic process, allowing them to move into the intima and build the plaque’s extracellular matrix [211]. Phenotypic modulation refers to phenotypic alterations that are important in vascular remodeling, which might result in atherosclerosis, hypertension, or diabetic macroangiopathy [212]. Lycopene prevents G1 phase cells from entering the S phase of the cell cycle [213], not by inhibiting matrix metalloproteinase [214]. It was also found that minimally oxidized LDL-C can cause VSMC phenotypic modification; to suppress this process, lycopene may inhibit oxidized LDL formation [215,216]. Direct binding to platelet-derived growth factor (PDGF) and reducing PDGF signaling [217] or acting as an antioxidant, since reactive oxygen species speed up the switch from contractile to synthetic phenotype [218], were observed to limit VSMC proliferation and migration. Lycopene also suppresses apoptosis in endothelial cells in vitro by interrupting the upregulation of p53 and caspase 3 mRNA, and prevents cluster of differentiation 14 (CD14) and toll-like receptor-4 expression in the endothelium membrane [219,220]. Circulating plasma lycopene is thought to protect against atherosclerosis (Figure 5), especially in smokers [221,222]. In high-fat diet rabbits, lycopene decreased the development of atherosclerotic plaques in the aorta and improved the lipid profile compared to the control group [223]. In hypertensive individuals, a short-term therapy with antioxidant-rich tomato extract (250 mg/day for eight weeks) can reduce blood pressure [224]. According to a research, lycopene consumption and carotid artery intima-media thickness, a risk factor for CVD, have an inverse association. [225].

### 5.2. Hypertension and Lycopene

A systolic blood pressure of more than 140 mmHg or a diastolic blood pressure of more than 90 mmHg, or both, is characterized as systemic arterial hypertension (HT), often known as high blood pressure [226]. As the silent killer, promoted by low-density lipoprotein cholesterol and smoking, it remains the third-leading cause of mortality from cardiovascular disease. Hypertension is also a major cause of stroke and renal failure. Hypertension is associated to oxidative stress and inflammatory processes [227,228]. Chronic hypertension can cause renal failure, left ventricular hypertrophy (LVH), chronic heart disease (CHD), heart failure (HF), peripheral vascular disease, stroke, and retinopathy.

**Figure 6:**
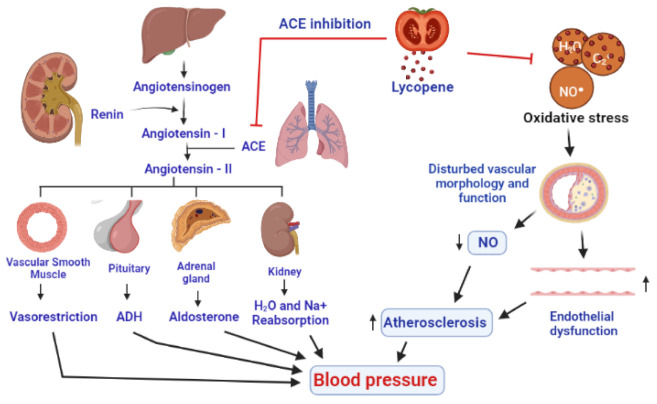
A presentation of antihypertensive effects of lycopene by inhibition of ACE (angiotensin-converting enzyme) and through an antioxidant activity to inhibit the action of ROS on the linings of the blood vessels, resulting in the improvement of nitric oxide levels and the functioning of endothelium. Eventually, a decrease in vasoconstriction, inhibition of antidiuretic hormone and aldosterone, and decrease in the reabsorption of water and Na^+^ by the kidneys decrease the blood pressure.

The pathophysiology of hypertension is highly complex and multifactorial. A complex interaction of environmental and pathophysiological variables that impact many systems have been reported to contribute to the development of hypertension. According to one opinion, as described by some research reports, hypertension is developed by the influence of oxidative stress. Oxidative stress makes changes in the structure and function of blood vessels by lowering the nitric oxide (NO), resulting in endothelial dysfunction and vascular cell proliferation, migration, and apoptosis, all of which promote hypertension [229]. The blood pressure-lowering effects of ROS scavengers, antioxidants, and NOX inhibitors also support the role of oxidative stress in the pathogenesis of hypertension [230]. NO is produced by endothelial cells that operate in coordination with prostacyclin to inhibit the production of adhesion molecules and aggregation of platelets. Hence, in the absence of NO, the components and mechanisms of atherosclerosis are promoted. The pathogenesis of hypertension is also well described by the renin-angiotensin system (RAS), which also provides the potential targets for the therapies against hypertension [230]. Angiotensinogen, a precursor of angiotensin, is produced in the liver, while renin is the enzyme produced by the juxtaglomerular cells of the kidneys, which catalyzes the first step in the processing of angiotensinogen into angiotensin I [231]. The angiotensin-converting enzyme (ACE), mainly produced in the lungs, is responsible for the conversion of angiotensin I into angiotensin II [232], which interacts with corresponding receptors and leads to the production of multiple biological molecules including ADH (antidiuretic hormone), aldosterone, and potent agents for vesorestriction from smooth muscle cells of vessels, adrenal, and pituitary glands. The combined effects of all these developments include the reabsorption of sodium and water by kidney, decrease in the amount of urine, narrowing of blood vessels, and subsequent onset of hypertension [233] (Figure 6).

Lycopene reduces oxidative stress indirectly increases nitric oxide (NO) generation in the endothelium, acting as an antioxidant and decreases blood pressure. After 6 weeks of tomato extract supplementation, a significant reduction in both systolic and diastolic blood pressure was found in 54 patients suffering from moderate hypertension, already using ACE inhibitors or calcium channel blockers, indicating a contributory role of lycopene in the management of hypertension [229]. In a meta-analysis, lycopene supplementation (above 12 mg/day) was found to lower systolic blood pressure in prehypertensive and hypertension patients, while it has shown no effect on diastolic blood pressure [234,235]. Lycopene can impede the angiotensin II by inhibiting the ACE [2]. Due to the antioxidant and anti-inflammatory properties, lycopene supplementation prevented changes in hemodynamic parameters, biochemical and inflammatory markers, apoptotic alterations, and reduced the extent of myocardial infarction. A study on 299 Korean men found that they had a significant reduction in their blood pressure [230], after 8 weeks of 15 mg/day lycopene supplementation [236] (Figure 6).

## 6. Conclusions

Lycopene has been shown to have a variety of biological effects in epidemiologic research and animal and cell culture investigations. These findings have prompted more research into the role of lycopene and its derivatives in the development of chronic illnesses. More research is certainly needed to identify and describe additional lycopene metabolites as well as their biological activities, which could provide vital insight into the processes behind lycopene’s positive benefits in humans, especially in terms of chronic disease prevention. Furthermore, higher lycopene consumption is linked to a lower risk of death from cardiovascular disease-, stroke-, and hypertension-related injuries. It will be determined in the future whether lycopene with increased bioavailability can maintain its antioxidant effect on lipoprotein oxidation and cardiovascular markers over time. Research with a longer duration of lycopene supplementation and a placebo control group are needed, as well as the correct amount, as there are a variety of studies demonstrating varying benefits with different doses, but no clear criteria to identify an accurate dose for hypertension and CAD patients. It is also crucial to see how this lycopene treatment affects other clinical manifestations of hypertension and coronary artery disease. Additionally, it is important to look into the effects of different lycopene isomers on oxidative and cardiovascular markers. Nonetheless, our findings imply that lycopene supplementation has a lot of potential in the treatment of cardiovascular disease and could be used to improve the inflammatory state and cardiovascular parameters in hypertension and coronary artery disease patients.

## Figures and Tables

**Table 1:** An illustration of the nutraceutical impact of lycopene against diseases.

Diseases	Issue	Dose of Lycopene	Subjected Time	Role/Activity of Lycopene	References
Cancer	Loss of gap junctional communication (GJC)	4–8 mg	3–12 months	Suppression of carcinogen formation.	[92]
		60 mg	9 weeks	GJC is boosted by a lycopene oxidation product.	[66,122]
		50 mg/kg	5 to 7 days	Metabolite of lycopene, can enhance connexln 43, which is linked to GJC.	[116,123]
	Apoptosis	10, 40, 120 mg/kg	9 weeks	Lycopene triggers apoptosis in cells.	[124]
	Melanoma	10 mg/kg 15 mg/day	5 weeks 12 weeks	Lycopene inhibits melanoma development.	[121,125]
	Mammary and endometrial cancer	76 to 154 mg	14 days	Lycopene inhibits the insulin-like growth factor 1 (IGF-1R) signaling pathway.	[125,126,127]
	Prostate and colon cancer	10–30 mg	3 to 5 days	Inhibit Ras signaling.	[26,128]
	ovarian cancer	20–40 mg/kg	18 weeks	Downregulation of STAT3 reduces tumor development.	[129]
	Lung cancer	5 mg/kg	16 weeks	Inhibits lung cancer.	[87]
	Oral cancer	5 mg/kg	16 weeks	ROS scavenger.	[87]
Vitamin A deficiency		10–2400 mg	90 days	Upregulate signaling pathways	[130]
Inflammatory Diseases	Brain tissue inflammation	50, 100, 150 mg/kg	24 weeks	Lycopene boosts antioxidant gene expression, inflammatory mediators.	[17,119]
		60 mg/kg	7 days	Lycopene helps to prevent inflammation by lowering the levels of plasma interleukin (IL)-6 and TNF.	[131,132]
Skin Diseases	Photodamage by UV-B	10 mg/kg	5 weeks	Lycopene inhibits epidermal ornithine decarboxylase. Prevents DNA damage.	[121,133]
	Atopic dermatitis (AD)	100 mg	7 days	The activation of nuclear hormone receptor signaling pathways.	[134].
	Photo aging	2.5–10 µM	24 h	Lycopene gel provided superior photoaging protection.	[135]
Cardiovascular diseases		2 mg/day	12–20 weeks	Lycopene reduces atherosclerotic plaques.	[136,137]
Bacterial infection		50, 100, 150 mg/kg	24 weeks	Lycopene stimulates immune response.	[119,138]
Age-related macular degeneration		4 mg/kg/day	10 weeks	Lycopene is capable to quench singlet oxygen in the eye.	[139]
Diabetes		10 mg/kg	5 weeks	Enhanced antioxidant enzymes, suppresses RAGE expression, increased NF-B expression.	[140,141]
Infertility		4–8 mg	3–12 months	Boosted antioxidants, reduced lipid peroxidation, decreased DNA damage in spermatozoa.	[92,142]
Neurobehavioral deficits and poorer cognition		10 mg/kg	15 days	Increased cognition, increased brain’s antioxidant, nitric oxide pathways are inhibited.	[143,144,145,146]
Alzheimer disease		30 mg	5 weeks	Reduced mitochondrial dysfunction, reduced inflammatory cytokine.	[147,148,149]
Parkinson disease		15, 30, 45 mg/kg/day	12 weeks	Induction of ROS and neurobehavioral deficits.	[150,151,152]
Bone diseases		50 mg/kg/day	10 weeks	Regulation of metabolism, osteoclast differentiation, osteoblasts upregulation.	[153,154,155]

## Data Availability

The data presented in this study are available in the paper.
